# Insights from animal models of bladder cancer: recent advances, challenges, and opportunities

**DOI:** 10.18632/oncotarget.17714

**Published:** 2017-05-09

**Authors:** Bincy Anu John, Neveen Said

**Affiliations:** ^1^ Department of Cancer Biology, Wake Forest School of Medicine, Winston-Salem, North Carolina, USA; ^2^ Department of Pathology, Wake Forest School of Medicine, Winston-Salem, North Carolina, USA; ^3^ Department of Urology, Wake Forest School of Medicine, Winston-Salem, North Carolina, USA

**Keywords:** bladder cancer, animal model, xenografts, carcinogen-induced, genetically engineered mice

## Abstract

Bladder cancer (urothelial cancer of the bladder) is the most common malignancy affecting the urinary system with increasing incidence and mortality. Treatment of bladder cancer has not advanced in the past 30 years. Therefore, there is a crucial unmet need for novel therapies, especially for high grade/stage disease that can only be achieved by preclinical model systems that faithfully recapitulate the human disease. Animal models are essential elements in bladder cancer research to comprehensively study the multistep cascades of carcinogenesis, progression and metastasis. They allow for the investigation of premalignant phases of the disease that are not clinically encountered. They can be useful for identification of diagnostic and prognostic biomarkers for disease progression and for preclinical identification and validation of therapeutic targets/candidates, advancing translation of basic research to clinic. This review summarizes the latest advances in the currently available bladder cancer animal models, their translational potential, merits and demerits, and the prevalent tumor evaluation modalities. Thereby, findings from these model systems would provide valuable information that can help researchers and clinicians utilize the model that best answers their research questions.

## INTRODUCTION

Bladder cancer is the most common malignancy of the urinary tract and the second most common malignancy of the urogenital tract following prostate cancer in the United States [1]. The American Cancer Society estimates 76,960 new cases and 16,390 deaths of bladder cancer in the US with male: female ratio of 3:1. These figures indicate that bladder cancer is now the fourth most common among men and the ninth most common in women (http://www.cancer.org). The primary risk factors for bladder cancer include environmental and occupational exposures to chemical carcinogens [2] as tobacco smoke/metabolites, aromatic hydrocarbons, house paints, fungicides, plastics, and heavy metals [2–4]. Also, *N*-nitroso compounds (NOCs) used in chemically preserved food products are associated with risk for bladder cancer [5]. A family history of bladder cancer is linked to two-fold higher risk, but bladder cancer affected families are not common and no high-penetrance genes have been identified [6–8].

**Figure 1:**
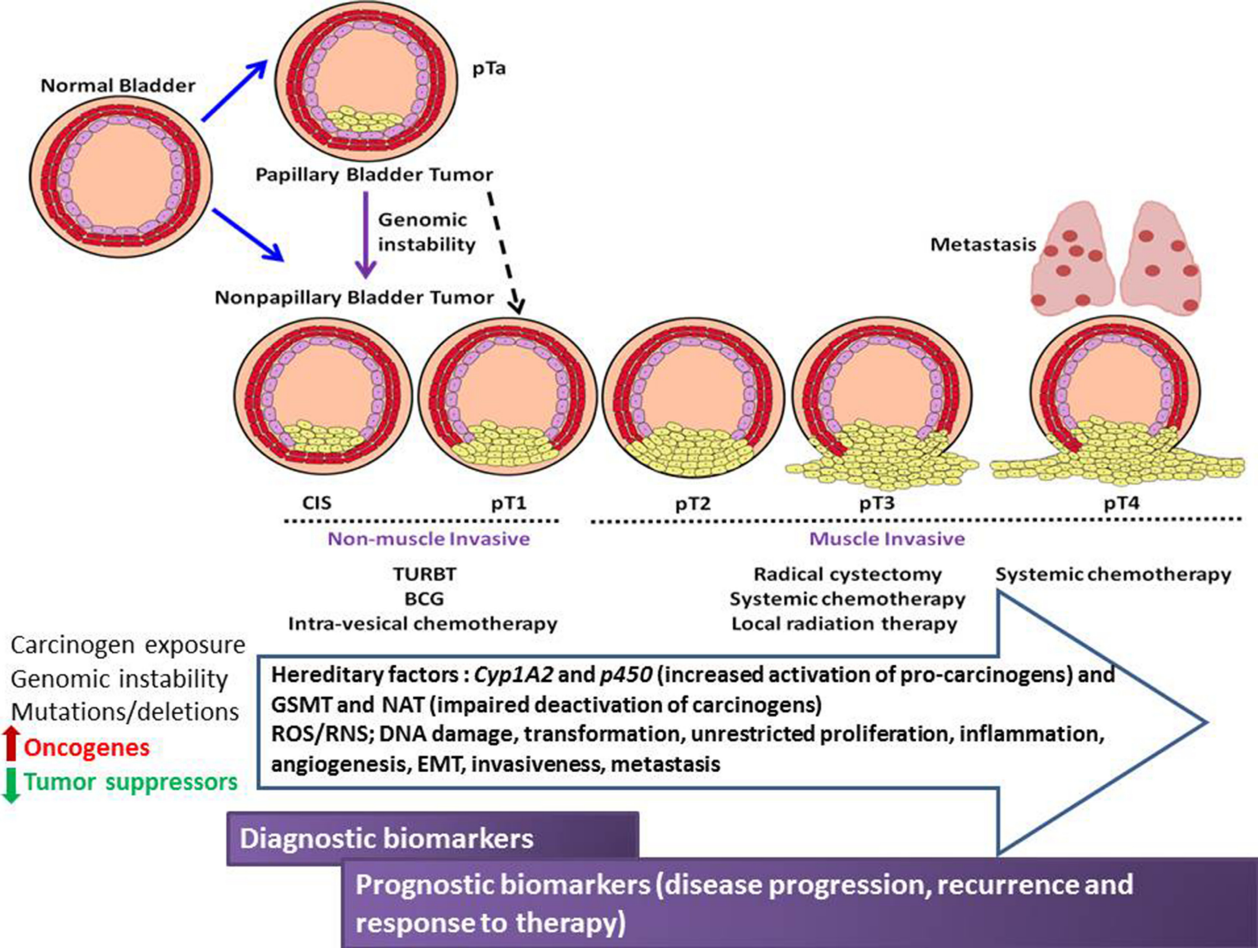
Schematic illustration of the pathological stages of urothelial cancer of the bladder, therapeutic intervention, environmental, genetic and molecular influences of urothelial carcinogenesis, progression and metastasis Opportunities for diagnostic/prognostic biomarker discovery are shown compared to disease stage.

Bladder cancer typically arises from the urothelium, the well-differentiated transitional epithelium that lines the urinary bladder. The majority of bladder cancers diagnosed in developed countries is of transitional cell histology and is known as urothelial carcinoma (UC). Bladder cancer develops via two clinically and pathologically distinct routes: papillary and non-papillary forms of disease [9, 10] (Figure 1). About 75–80% of urinary bladder tumors are superficial papillary lesions known as non-muscle invasive urothelial cancer (NMIUC) and are referred to as low-grade intra-urothelial neoplasia. These tumors can be multifocal and may recur after local excision, but typically they do not invade the bladder wall or metastasize. Development of low grade lesions is associated with molecular aberrations in the oncogene RAS, FGFR3, and deletions of 9q [11]. Twenty to twenty-five percent of patients are present with solid, non-papillary tumors that invade the detrusor muscle which is called muscle invasive urothelial cancer (MIUC). These originate from carcinoma *in situ* (CIS) or severe dysplasia. These tumors have a high tendency to give rise to distant metastases. Hence, they are referred to as high-grade intra-urothelial neoplasia and are associated with alterations in p53, retinoblastoma (Rb), and PTEN [12]. Treatment of bladder cancer and the efficacy of such treatment vary profoundly depending on the clinical stage and associated risk factors [13–15]. The primary treatment of NMIUC is transurethral resection of bladder tumors (TURBT) [16] followed by intravesical therapy either by instillation of mitomycin C or with Bacillus Calmette-Guerin (BCG) [17, 18]. These treatments result in longer disease-free survival in patients with low-grade disease, but recurrence is common in patients with high-grade disease with progression to locally advanced muscle invasive and metastatic disease [17, 18]. High risk patients or those with recurrence after TURBT are treated with several cycles of BCG, which promotes immunoreaction against cancer cells [19]. BCG treatment requires frequent surveillance over long periods of time with adverse effects including severe urocystitis or systemic tuberculosis [20, 21]. Clinical management of MIUC primarily focuses on cystectomy (surgical removal of bladder) with neoadjuvant or adjuvant chemotherapy and/or radiation therapy for selected individuals [22, 23]. Metastatic bladder cancer generally treated by a multidrug chemotherapy regimen consisting of methotrexate, vinblastine, adriamycin, and cisplatin (MVAC) or, alternatively, gemcitabine plus cisplatin (GC) [16]. In addition to the side effects of chemotherapy, bladder cancer is highly resistant and upon relapse, effective treatment options are limited [24]. There has been no new approved treatment for bladder cancer in the past 30 years [25, 26]. Therefore, there is a dire unmet need to better understand the pathobiology of bladder cancer, identify diagnostic and prognostic biomarkers of disease progression and develop new therapeutics. To achieve these goals, several experimental models of bladder cancers are developed and undoubtedly, they present useful tools for molecular and functional analyses, and preclinical pharmacological assessment.

### Bladder cancer *in vivo* models

An ideal animal model of bladder cancer should recapitulate its human counterpart with similar histopathological features, natural course of tumor growth and progression. Most importantly, these models should possess a high reproducibility, predictive and translatability value to allow mechanistic, chemo-preventive and therapeutic studies that can be furthered into human clinical trials. The most commonly used species for animal research are small rodents, such as mice and rats. Rodents have a lower urinary tract that is homologous to that of humans. Also, bladder cancer is not very common in rodents unless they are induced by a chemical carcinogen [27] or oncogenes [28]. These small animals can be easily housed, maintained at a low cost and are useful in generating valuable information regarding bladder carcinogenesis. Mouse strains such as C57B6, BALBC, and ICR, and rat strains such as Wistar, Sprague-Dawley and Fisher are most commonly used for bladder cancer research [29]. Some rat strains such as Brown Norway and DA/Han show high incidence of spontaneous bladder tumors, and thus can be used as experimental models without treatment with chemical carcinogens [30]. Larger animals such as dogs, rabbits, guinea pigs and hamsters have all been used in the past as bladder cancer models [31]. However, their use is limited due to financial and ethical constraints.

**Figure 2:**
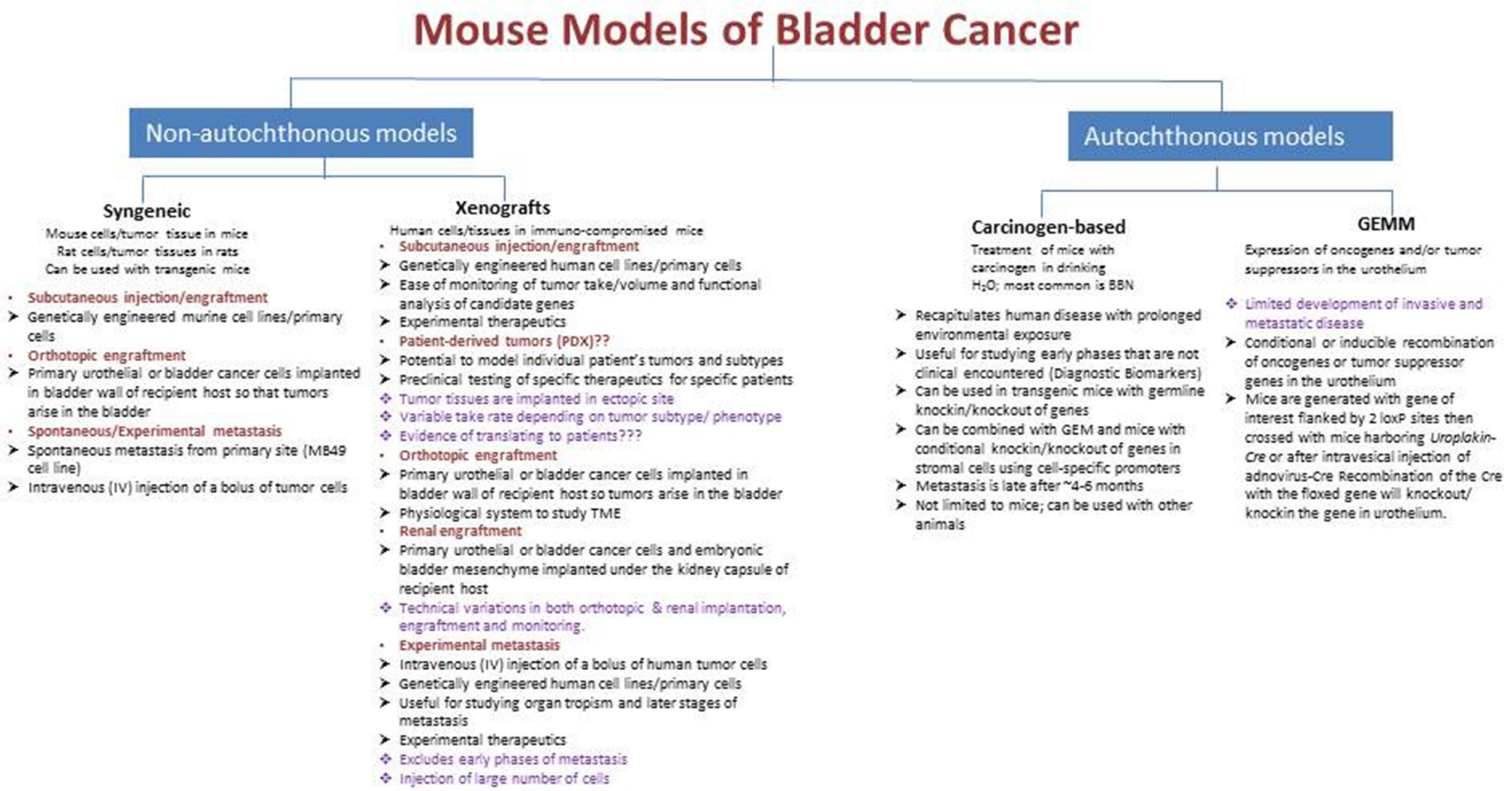
Summary of the available mouse models of urinary bladder cancer

Animal models of bladder cancer (Figure 2) can be categorized as autochthonous (spontaneous) and non-autochthonous (transplantable). The first are either chemically induced models or genetically engineered models. The transplantable models can be further sub-classified as syngeneic (murine bladder cancer cells implanted into immunocompetent or transgenic mice) and xenografts (human bladder cancer cells implanted into immune-deficient mice). These models can be further divided based on the site of tumor as orthotopic (tumor growth occurs within bladder) and heterotopic (tumor growth occurs outside of the bladder).

### Autochthonous (spontaneous) models

#### Carcinogen induced model

Bladder cancer is caused by continuous exposure to chemical carcinogens such as tobacco, aromatic amines and chlorinated hydrocarbons [32]. Therefore, it is essential to examine the relationship between chemical carcinogenesis and the development of urothelial carcinoma of the bladder. The first urothelial carcinogenesis model was induced in rats [33–35], thereafter several carcinogens were developed in various species, including mice, rats, and dogs [36]. Urothelial carcinoma can be induced predominantly in rodents with the use of several chemical carcinogens. The major chemical urothelial carcinogens are *N*-Butyl-*N*-(4-hydroxybutyl)nitrosamine (BBN), N-[4-(5-nitro-2-furyl)-2-thiazolyl]formamide (FANFT), and N-methyl-N-nitrosourea (MNU). Most of these carcinogenic agents have aromatic amine components. The carcinogen models develop over a prolonged period of time recapitulating the prolonged human exposure.

BBN was first identified as a bladder carcinogen in rodents [34, 37] and is detected in tobacco smoke and environmental and infectious metabolites [29]. BBN-induced cancer models can be established either orally by adding it to drinking-water and is degraded to N-butyl-N-(3-carboxypropyl)-nitrosamine, which has carcinogenic effects on the urothelium when cleared in the urine [29]. BBN-exposed mice develop various pathological features, including hyperplasia, dysplasia, CIS, and invasive tumors, as well as metastases that are histologically and genetically similar to human bladder tumors arising from extensive tobacco use [29]. In line with this, Williams *et al*. [38] compared gene expression profiles of urothelial carcinoma for three different species: mouse, rat, and human. Several human genes homologous to those differentially expressed in carcinogen-induced rodent tumors were also differentially expressed in human bladder cancer and were associated with progression to muscle-invasive disease. The overall gene expression profiles of rodent tumors tracked with those of invasive human tumors rather than those of non-muscle-invasive tumors. The cell cycle associated genes such as cell division cycle 20 (*CDC20*), cell division cycle 2 (*CDC2*), cyclins D1 and B2 (*CCND1* and *CCNB2*), mitotic arrest–deficient 2, *Saccharomyces cerevisiae*, homolog-like 1 (*MAD2L1*), and cyclin A2 (*CCNA2*) were primarily differentially expressed between normal and cancerous urothelium in all three species [38]. Furthermore, Lu *et al*. [39] reported that changes in several protein/mRNA levels were consistent in BBN murine model versus human bladder cancer, thereby reinforcing the reliability of this model.

BBN-induced rodent tumors, predominantly mouse tumors, have *p53* mutations, or mutations in genes related to the *p53* pathway, especially in high-grade tumors [37, 40]. *H-Ras* mutations are observed occasionally in mouse and rat models, although BBN-induced carcinogenesis occurs more efficiently in *H-Ras* transgenic mice. BBN-induced tumors show high levels of Epidermal growth factor receptor (EGFR) [37, 41]. *Nrf2^−/−^* null mutation in both *Nrf2* alleles and *p27^kip^* null mutation in both *p27* alleles resulted in higher sensitivity to BBN than the wild type mouse [42–44].

BBN chemical carcinogenesis model in transgenic mice proved to be a powerful tool to identify the mechanism of action of tumor suppressor and oncogenes as well as the pre-neoplastic lesions that are not clinically encountered [45]. In this respect, the role of the matricellular glycoprotein, secreted protein acidic and rich in cysteine (SPARC) in the pathobiology of bladder cancer was reported [45]. This model showed that genetic ablation of SPARC augmented BBN-induced bladder carcinogenesis, progression and metastasis. The authors identified that SPARC restrains urothelial carcinogenesis, progression and metastasis through an effect on cancer cells inhibiting carcinogen-induced cell cycle deregulation and on stromal cell plasticity, inhibiting their acquisition of a pro-inflammatory cancer associated phenotype. In another study, utilizing *Rgs6^−/−^* mice, the role for *RGS6* as a tumor suppressor was reported as they displayed accelerated pathological lesions than *Rgs6^+/+^* mice [46]. The investigators identified RGS6 as a tumor suppressor modulating ATM/p53 and RASSF1A, the two critical signaling pathways that are often dysregulated in urothelial carcinoma. BBN-induced rodent models were analyzed by genome-wide analysis of copy number aberrations in bladder lesions [47]. The tumor promoting effect of phospholipase Cε (PLCε) was also studied using BBN in PLCε knockout mice [46]. The tumor promoting effect of PLCε was demonstrated by a decreased incidence of ensuing urothelial lesions in PLCε knockout mice compared to their wildtypes as well as a significant downregulation of PLCε downstream targets such as VEGF-A and COX-2 [46]. Array CGH revealed that several mouse chromosome regions, including the *Cyp2a5* and *Cyp2a22* loci on mouse chromosome 7qA3, were amplified in invasive bladder cancer. Interestingly, the human *CYP2A6* gene, ortholog of the mouse *Cyp2a5* gene was found to be upregulated in human cell lines and in resected human bladder cancer specimens [47]. BBN model can be also used to study the impact of conditional cell/tissue specific knockout/knockin of genes; for example, the effect of conditional knockout of estrogen receptor α and β [48] as well as androgen receptor [49]. Moreover, this model was used to evaluate the preventive and therapeutic efficacy of chemotherapeutics [46, 50–52].

MNU is another genotoxic carcinogen that acts directly on the urothelium by intra-vesical instillation and leads to constitutive DNA methylation [53]. The primary advantage of this model is that papilloma and carcinoma appear after 12 weeks and MNU is the only carcinogen to produce bladder cancer at a single dose [35]. However, MNU is intrinsically unstable and should be stored at a low temperature and protected from light. Therefore, it may result in experimental inconsistencies due to its decomposition and altered carcinogenic potency over time.

FANFT is an indirect chemical carcinogen that stimulates the bladder mucosa, and the bladder tumors mostly develop into transitional cell carcinoma (TCC) after it is fed to rodents for 5 to 8 months [54]. FANFT is not currently common as it is identified as an environmental pollutant and is hazardous for human health.

### Genetically engineered models

The transgenic mouse, or genetically engineered mouse (GEM), are useful research systems that are engineered to carry cloned oncogenes or lack tumor-suppressing genes, and allows investigation of human disease associated genetic abnormalities *in vivo*. They allow studies of individual as well as multiple mutational events which eventually orchestrates bladder tumorigenesis. Several methods are employed to generate GEM models. Mice with germ line deletion may not allow investigation of the gene function if knockout leads to premature death or embryonic lethality and do not allow for the clear distinction of the tissue/cell-specific contribution of a given gene in the disease. An approach that avoids these limitations is a conditional gene knock-in or knockout involving the *Cre-loxP* system, which allows for studying gene function in specific cell and tissue types [55]. Urothelial-specific *Cre* system is available and is typically used to selectively achieve gene knockin/knockout in the bladder epithelium. The Cre-*loxP* system is based on the bacteriophage P1 wherein *Cre* recombinase acts on palindromic sequences called *loxP* sites that have been genetically engineered into the specific sites in the mouse genome. *Cre* recombinase can then excise the genomic sequence between two *loxP* sites. Therefore, mouse alleles containing *loxP* sites can be used for introducing mutations, or for a temporally or spatially controlled gene knockout. Majority of the bladder cancer GEM models have used the mouse *Uroplakin II* (*UpkII*) promoter which is the 3.6 kb 5′-upstream sequence of mouse *UpkII* gene [56]. Uroplakins are membrane integral proteins that constitute the major differentiation products of the urothelium. Zhang *et al*. demonstrated that SV40 (Simian virus 40) mice generated using *UPII* promoter developed CIS urothelial carcinoma with low copy number of SV40T transgene and those with high copies developed CIS along with invasive and metastatic transitional carcinoma [57]. Ayala de la Pena and colleagues [58] developed the promoter known as *Upk II* which was used to generate mice expressing the oncogene *SV40* large T-antigen that can inactivate *p53* and retinoblastoma (*Rb*) proteins [58]. GEM models are widely used for investigating specific gene functions including, *H-RAS, p53, RB, PTEN*, fibroblast growth factor receptor (*FGFR*), and epidermal growth factor receptor (*EGFR*), in the development of bladder cancer. *H-RAS* was amongst the initially identified oncogenes in human bladder cancer [59]. *RAS* activation can occur through overexpression of *H-Ras* in the urothelium, which occurs in more than 50% of bladder cancers [60]. A transgenic model was developed to allow targeted expression of a constitutively active *H-Ras* in the urothelium using the *UpkII* promoter [44]. This mutation resulted in early onset urothelial hyperproliferation, which progressed to low-grade, papillary, non-invasive tumors. This study revealed a strong relation between tumor latency and *H-Ras* copy number. Mice with low-copy (one or two copies) number of genes showed shorter tumor latency and developed non-invasive lesions compared to mice with high-copy numbers (above 30 copies). He *et al*. developed a conditional compound mice expressing oncogenic HRAS and lacking p53 gene expression. This model resulted in CIS and muscle-invasive urothelial carcinoma with focal squamous differentiation similar to human counterpart. This study suggested that the RTK/RAS pathway activation along with p53 deficiency can be used as a combinatorial therapeutic marker to indicate urothelial carcinoma progression [61]. *FGFR3* is another gene that is often associated with low grade bladder cancer. Ahmad *et al*., [62] developed a model using *UpkII* promoter to induce expression of mutated *Fgfr3* to the mouse urothelium. Mutated *Fgfr3* alone was insufficient to induce urothelial tumorigenesis. When *Fgfr3* mutations were introduced along with overexpression of *K-Ras* or *β-catenin* inactivating mutations, mice developed tumors indicating that *Fgfr3* cooperates with other mutations to drive tumorigenesis [42, 62, 63]. In addition, mutations and/or deletions in the p53 gene are among the most common genetic alterations found in human bladder cancer [11, 25]. Consistently, He and colleagues [64] demonstrated that urothelial-specific deletion of both copies of *Rb* did not augment urothelial proliferation, but conversely, this deletion fostered activation of the *p53* pathway and led to apoptosis. Double-mutant mice that were null for both *p53* and *Rb* did not exhibit accelerated tumorigenesis, though they became extremely sensitive to BBN induced carcinogenesis and invasiveness [64]. This study suggested that genetic ablation of both *p53* and *Rb* is essential for the progression of UCC, but insufficient to initiate invasive UCC by itself. Allelic loss of *Tp53* alone was sufficient to drive tumorigenesis but not invasiveness of urothelial cancer [43, 65]. The *PTEN-PI3K-AKT* pathway has been implicated in bladder cancer and reports suggest that deletion of *PTEN* occurs frequently in invasive UCC [25, 42, 66]. Studies crossing *Uroplakin-Cre* mice with reported *Pten* floxed mice revealed the co-operation of activation of *β-catenin* and with *Pten* deletion to drive urothelial carcinogenesis [42]. Expression of the activated form of *β-catenin* led to the formation of localized hyper-proliferative urothelial lesions by 3 months, which did not progress to malignancy. Targeted deletion of *Pten* and expression of activated *β-catenin* caused papillary UCC that exhibited increased pAKT signaling and were dependent on mammalian target of rapamycin (mTOR). Importantly, in human UCC, there was a significant correlation between high levels of *β-*catenin and pAKT (and low levels of *PTEN*). Moreover, targeted deletion of both *p53* and *Pten* [67] using an adenovirus expressing *Cre* recombinase delivered directly into the bladder of mice with floxed alleles of *p53* and *Pten* (*p53^fl/fl^Pten^fl/fl^*), developed bladder tumors with 100% penetrance after 24 weeks, with metastasis to local lymph nodes and distant sites, including the spleen, liver and diaphragm. This work strengthened the notion that urothelial bladder cancer is a complex disease with no single driver mutation, but requires multiple mutations to induce bladder tumorigenesis. Yoo *et al*, [68] developed a GEM model by deleting exons 4–5 of the *Pten* *gene* using the *FabpCre* system, in which the expression of *Cre* recombinase was placed under the control of transcriptional regulatory elements from a fatty acid-binding protein (*Fabp*) gene. Crossing *Fabp-Cre* mice with *Pten^fl/fl^* mice resulted in loss of *Pten* in all of the cell layers of the urothelium as well as prostate, vagina and intestine. Of note that *Fabp-Cre* mice unexpectedly developed lesions in the terminal small intestine, cecum, colonic mucosa and other pelvic organs including urinary bladder [69]. *Fabp-Cre*-*Pten^fl/fl^* mice developed urothelial hyperplasia and UCC by 13.5 months (∼44 weeks) of age with decreased levels of p27, increased levels of p21, and a decrease in proliferation rates in the urothelium [68]. These observations were distinctive from concurrent prostate lesions. Thus investigators concluded that deletion of *Pten* induces distinct tissue-specific pathways that influence tumor and progression [68].

Another approach to conditionally manipulate genes in the urothelium is the direct injection of adenovirus-*Cre* in the bladder lumen. This method has been used to investigate the roles of the main driver mutations as *K-ras*, *p53, Pten* and *Rb* in the temporal and spatial development of urothelial cancer [67, 70, 71]. These studies further identified therapeutic targets downstream of the aforementioned mutations. Of note, Yang and colleagues [71] observed that the simultaneous inactivation of *p53* and activation of *K-ras* induces quick formation of spindle-cell sarcoma in the soft tissues adjacent to the bladder but slow formation of urothelial hyperplasia inside the bladder. These results strongly suggest that the effect of oncogene regulation to produce either hyperplasia or carcinogenesis greatly depends on the tissue type. Most importantly, studies with the GEM models that recapitulate disease progression allowed the preclinical validation of the key pathways involved in urothelial cancer progression from NMI to MI disease [26, 67, 70, 72] and paved the way to clinical trials for intra-vesical delivery of single or combinatorial chemotherapeutics to prevent disease progression in high risk patients with early stage disease [26].

### Non-autochthonous (transplantable) models

The transplantable models (xenografts and syngeneic) can be categorized as heterotopic and orthotopic models based on site of tumor cell implantation. It is essential to choose representative and reproducible cell lines for transplantable models. A large number of human and rodent urinary bladder cancer cell lines are available representing different origins, grades and stages of urothelial carcinoma, and mirror many of the genetic, morphologic, and gene expression alterations observed in human urothelial carcinoma [73]. Several cell lines were established from invasive and metastatic tumors, which are advantageous in the investigation of late tumor progression and metastatic lesions [74, 75] (Table 1). Important resources with detailed information regarding genetic alterations in cell lines are the Catalog of Somatic Mutations in Cancer (COSMIC) at the Sanger Institute (Cambridge, United Kingdom) [76], the Cancer Genomic Atlas (TCGA) for tumor samples [25], the Cancer Cell Line Encyclopedia (CCLE) [77], and the Genomics of Drug Sensitivity in Cancer [48]. It is imperative that cell lines are authenticated to ensure the use of correctly identified uncontaminated cell lines in research studies. Therefore, it is recommended to purchase cell lines from authenticated cell repositories. Identity verification with Short tandem repeat (STR) profiling establishes a DNA fingerprint for every human cell line and may be used as a record of the line. STR profiling uses multiplex PCR to simultaneously amplify several polymorphic markers in the human genome. Each cell line exhibits a pattern of repeats which constitutes the unique STR identity profile [78].

**Table 1:** Genetic characterization of major oncogenes and tumor suppressor genes in human bladder cancer cell lines

Cell Line	Source	Gender	Species	Histology	PIK3CA	HRAS	KRAS	NRAS	TERT	TP53	PTEN	ERBB2	FGFR3	KDM6A
253J	UT	F	Human	TCC	p.E545G	WT	WT	WT	WT	WT/N		WT	WT	
253J-BV	UT	F	Human	TCC	p.E545G	WT	WT	WT		WT	WT		Deletion	
5637	UT	M	Human	TCC		WT	WT	WT	Mut	p.R280T	WT	p.S310F	Deletion	WT
575A	UT	M	Human	TCC	WT					WT			WT	
639V	UT	M	Human	C	p.A1066V	WT	p.G12D	WT/H131R	Mut	p.R248Q	p.R173C p.R130Q		p.R248C	WT
92-1	UT	F	Human	TCC	WT	WT	WT	WT	Mut				WT	
96-1	UT	M	Human	TCC	WT	WT	WT	WT	Mut			WT	WT	
97-1	UT	M	Human	C	WT	WT	WT	WT	WT	WT	WT	WT	WT	
97-18	UT	Y	Human	TCC		WT	WT	WT	Mut			WT	WT	
97-24	UT	Y	Human	TCC	WT	WT	WT	WT	Mut				WT	
97-7	UT	Y	Human	C	WT	WT	WT	WT	Mut				p.S249C	
BC61	UT	Y	Human	TCC	WT	WT	WT	WT		WT			p.G370C	
HT1197	UT	M	Human	C	WT	WT	WT	WT						
HT1376	UT	F	Human	TCC	p.H694Y	WT	WT	WT	Mut	p.P250L				
HU456	UT	M	Human	C		p.G12S			WT					
J82	UT	M	Human	TCC	p.P124L	WT	WT	WT	Mut	p.K320N p.E271K			p.K652E	
JON	UT		Human	TCC	WT	WT	WT	WT	Mut				WT	
KK47	UT	M	Human	C		WT	WT	WT	WT	N				
MGH-U3	UT	M	Human	C	WT	WT	WT	WT	Mut	WT/N			p.Y373C	
MGH-U4		M	Human	C	p.H1047R				Mut	WT/N			WT	
PSI	UT	M	Human	C		WT	WT	WT	Mut	WT				
RT4	UT	M	Human	TCC	WT	WT	WT	WT	Mut	WT		WT	WT/Amp	WT
SCaBER	UT	M	Human	TCC	WT	WT	WT	WT	Mut	p.R110L			Deletion	
SW-1710	UT	F	Human	TCC	WT	WT	WT	WT	Mut	p.R273C			Deletion	
SW-800	UT	M	Human	TCC	WT	WT	WT	WT	Mut	WT				
SW-850			Human	TCC	WT	p.G12V	WT	WT						
SW-780	UT	F	Human	TCC	WT	WT	WT	WT	Mut	WT				
T24	UT	F	Human	TCC	WT	p.G12V	WT	WT	Mut	p.Y126				p.E947
TCCSUP	UT	F	Human	TCC	WT	p.E545K	WT	WT	Mut			WT	WT	WT
UMUC-1	UT	M	Human	TCC	WT	WT	WT	WT	Mut	p.P152S				p.Q1281
UMUC-2	UT	M	Human	TCC	WT	WT	WT	WT	Mut					
UMUC-3	UT	M	Human	TCC	WT	WT	p.G12C	WT	Mut				Deletion	p.L1348R
VM-CUB-1	UT	M	Human	TCC	p.E542K p.E674Q					p.R175H		p.S653C		p.I1289N

C, Carcinoma; TCC, transitional cell carcinoma; F, female; M, male; UT, urinary tract; WT, wild type; Mut, Mutant; N, copy number neutral;

Y, Y chromosome detected.

### Orthotopic models

Orthotopic models allow evaluation of tumor behavior in an organ-specific microenvironment. These models can be established by injecting urothelial cancer cells into the bladder lumen of recipient hosts. Transitional cells preferentially establish on an altered urothelial surface. For this purpose, the natural protective epithelium lining the surface of the urinary bladder has to be damaged to facilitate tumor cell adhesion. The mechanical damage can be achieved by electrical cauterization and epithelial abrasion [79–85] as well as chemical denudation with HCl [86, 87], N-methyl-N-nitrosourea [88], silver nitrate [80] followed by tumor cell instillation.

Several factors determine the efficient tumor take rate, such as the bladder preconditioning, cell concentration, the instillation volume and the tumor cell dwell time in bladder [89]. An instillation volume of 50–100 μL is typically used with mice weighing < 30 g. Higher volumes can result in peri-urethral leakage and reflux to the upper urinary tract. Therefore, it is highly suggested to ensure complete emptying of the bladder before instillation as it prevents over-distension of the mouse bladder [89]. Also, it has been previously reported that an increase in the tumor cell dwell time augments duration for which the cancer cells are in contact with the bladder mucosa, resulting in an increase in the tumor inoculation rate [90, 91]. Other than technical limitations, orthotopic models only metastasize to local lymph nodes and do not allow studies of metastasis or recurrence.

### Heterotopic transplantable models

These imply injecting/transplanting rodent or human bladder cancer cells or tissues in a rodent in an ectopic site other than tissue of origin. Thus, these models can be syngeneic (rodent cells/tissue in immunocompetent or transgenic rodent) or xenografts (human cells/tissue in immuno-deficient mouse). These models are adopted especially if the original site is not technically convenient for inoculation such as the urinary bladder. These models are easy to establish, easy to manage, cost-effective and are widely used in mechanistic studies as well as in evaluating the efficacy of novel therapeutic agents [92]. Several transplantable models are used in complementary way to study different aspects of tumor growth, and metastasis as well as the effect of tumor-stromal interactions [26].

### Syngeneic models

Syngeneic models are generated by implanting rodent bladder cancer cells or tissues into syngeneic, immunocompetent or transgenic animals. Rodent bladder cancer cell lines such as AY27 and MBT2 were initially induced by administering C3H/He mouse and Fischer 344 rat strains with FANFT) [86, 93], whereas MB49 was induced by feeding 7,12-Dimethylbenzanthracene to the C57BL/6 mouse strain [94]. Implantation of urothelial cancer cells in syngeneic hosts can be orthotopic (intravesical or heterotopic (subcutaneous, renal capsule implantation or experimental metastasis model). For instance, the use of the immunocompetent host in syngeneic models allows for the study of intravesical BCG treatment or gene therapy [79, 81, 95–100]. The murine MB49 bladder tumor model is quite similar to human bladder cancer which makes it an interesting model to study novel gene- and immunotherapies. Loskog *et al*. generated a subcutaneous mouse model using MB49 cells in C57BL/6 female and male mice [97]. Loskog et al. generated a MB49 metastatic tumor model using stably transfected luciferase MB49 cells, which could be monitored by *in vivo* bioluminescence. Another spontaneous metastatic model using syngeneic MB49 cell line revealed the significance of vasoconstrictor protein endothelin-1 (ET-1) in the early-establishment of metastasis. This study revealed that circulating tumor cells generate a strong inflammatory response in the lungs mediated by the ET-1/endothelin-1 receptor A (ET_A_R) axis and this allows metastatic colonization. Also, the pharmacologic inhibition of ET_A_R by ZD4054 prior to injection of tumor cells significantly decreased the early inflammatory response as well as the development of lung metastases. Therefore, this spontaneous metastatic model enabled us to determine that tumor ET-1 expression and ET_A_R activity are essential for metastatic lung colonization but their functional role is less significant in established primary or metastatic tumors. These data provide a major evidence that preclinical evaluation of new therapeutics should be done in the adjuvant setting using models of metastatic colonization [101]. The significance of tumor expression of the proteoglycan versican and chemokine CCL2 (also known as MCP1) in promoting bladder cancer lung metastasis was investigated using syngeneic MB49 metastatic model [102]. The expression of isoforms V1 and V3 of the proteoglycan versican is reduced by RhoGTP dissociation inhibitor 2 (RhoGDI2), which subsequently suppresses lung metastasis post tail-vein injection in *Ccl2^−/−^* and *Ccr2^−/−^* mice. Our findings emphasized the significance of CCL2 and CCR2 in GDI2-versican–mediated lung metastasis [102]. Additionally, using a syngeneic model of spontaneous metastasis in which SPARC-proficient MB49 cells were injected SC in *SP^−/−^* and *SP^+/+^* mice, host-SPARC not only inhibited the *in vivo* growth, invasiveness, angiogenesis and inflammation of primary tumors, but also mitigated spontaneous lung metastasis through inhibition of the pre-conditioning of the inflammatory pre-metastatic and metastatic lung niche [45]. Syngeneic models are both time and cost-effective, and reproducible. These model systems proved to be very useful in developing and validating new therapies at targeting the multistep cascade of tumor growth, progression, spontaneous metastasis and organotropism.

### Xenograft models

Xenografts imply the implantation of human tumor cells or tissues in immunocompromised mice. Xenografts can be subdivided into: orthotopic (intravesical) or heterotopic (subcutaneous, renal capsule implantation, or intravenous or intracardiac for experimental metastasis).

### Orthotopic xenografts

Chade *et al*. [80] described the use of intravesical 0.2% trypsin before treatment for 30 min, and mechanical bladder injury immediately before cancer cells are instilled for 3 h. Microscopic examination of the bladder 10 days after implantation revealed bladder tumors in 80–100% of mice, but the procedure-related death was high. Jager *et al*. [103] developed a novel high precision approach for orthotopic xenograft implantation. Bladder cancer cell lines such as UMUC1, UMUC3, and UMUC13 were inoculated into 10-week old athymic nude mice by percutaneous injection under ultrasound guidance. This model enabled monitoring of tumor volume, measuring *in vivo* tumor perfusion with microbubble contrast agents, and injecting therapeutic agents into the tumor under ultrasound guidance. The orthotopic tumors exhibit an increased microvessel density, high growth factors expression and proteolytic enzyme activity compared to those of the subcutaneous tumors. Moreover, rodents have a lower urinary tract which is comparable to humans, and neoplasms in the bladder are morphologically very similar, with a similar phenotype to human urinary carcinoma with respect to tumorigenesis and gene expression. However, orthotopic model establishment techniques are confounded by technical variability, with variable outcomes, complications and success.

### Subcutaneous tumor xenografts

Typically, human cancer cells are implanted in the flank or hind leg of immune-deficient mice. The subcutaneous model is the most commonly adopted model. This model is easy to establish, easy to manage, cost-effective and is used in mechanistic studies, and in evaluating the efficacy of novel therapeutic agents [92]. Another advantage of subcutaneous tumors is that they can be used to study local recurrence after excision. However, since subcutaneous tumors are not established at the original site, the urinary bladder, they do not recapitulate the real tumor microenvironment. In addition, subcutaneous xenografts do not metastasize, thus, cannot be used for studying some aspects of tumor biology specifically metastasis [104–106].

### Experimental metastasis models

They are used to study the mechanism of metastasis after tail vein or intracardiac injection of human cancer cells directly into the circulation. The inoculated cells require an initial adaptation phase in which the tumor cells acclimate to a new microenvironment. Cells that survive the turbulence in circulation grow and metastasize in the distant organs such as the lungs [107, 108]. The developed metastases can be isolated and cultured *in vitro* as metastatic derived isogenic cell clones that exhibit higher tumorigenic and metastatic potential compared with the parental cell line [107, 108].

### Tissue recombination models

Tissue recombination involves the isolation of fetal bladder mesenchyme from rats and then recombination with genetically altered human cell lines [67, 109]. It is possible to isolate mesenchymal tissue (and urothelium) from transgenic mice, enabling investigators to study the role of specific stromal contributions in bladder cancer progression. Another study reported the use of RT4 urothelial carcinoma cell line for tissue recombination experiments to identify the implications of p53 alterations and PTEN loss in urothelial carcinoma [67]. DeGraff *et al*. determined the influence of decreased FOXA1 expression for urothelial tumorigenicity using the tissue recombination system [109]. They used the non-tumorigenic RT4 cell line engineered to exhibit a low expression of FOXA1 and conducted tissue recombination experiments to determine the influence of decreased FOXA1 expression on bladder cancer cell proliferation. The resultant recombinants demonstrated enhanced RT4 proliferation and tumor volume. Majumdar *et al*. [110] identified the important role for endophilin A1 or SH3 domain containing GRB2 Like 2 (Sh3gl2) in bladder cancer using this model with RT4 depleted of Sh3gl2. They demonstrated that Sh3gl2 expression significantly declined in early-stage as well as in advanced bladder cancer, and silencing Sh3gl2 increased tumor volume. Therefore, this model enabled to understand the role of Sh3gl2 in bladder cancer as a predictor of the therapeutic response to RTK inhibitors.

The primary advantages of tissue recombination model include its relative cost-effectiveness, engineering flexibility and its ability to allow investigators to determine the impact of genetic alterations in tissue differentiation milieu. Tissue recombination is an efficient technique that allows testing the *in vivo* functions of different oncoproteins or tumor suppressors. These models offer an advantage over *in vitro* cell culture systems as they allow the determination of the influence of tumor microenvironment and facilitate the identification of stromal targets in cancer therapy. However, this model cannot recapitulate all aspects of the genetics, tumor heterogeneity, and complex etiology of bladder cancer. Also, the chances of discrepancies introduced through physical dissociation, lentiviral transduction, and transplantation could be high. Tissue recombination is a multistep approach which can be technically demanding and calls for enormous attention at all steps to avoid variability.

### Patient-derived xenograft (PDX) models

PDX models involve the direct transplantation of primary human tumor tissues from patients into immune-deficient mice. This model precludes the use of highly passaged cell lines [111] and allows studying relative contributions of the stroma and urothelium, as it realistically restores the original environment of the human tumor in a mouse. In 1979, the earliest attempts were made for establishment of bladder cancer xenograft models by implanting patient-derived tumor tissues [112–115], and the characterization was restricted to phenotypic analyses, such as Hematoxylin and Eosin (H&E) or immunocytochemical staining, without molecular or genomic analyses. Park *et al*. [116] developed a bladder cancer PDX model and were the first to use established genomic analyses techniques such as short tandem repeat genotyping, mutational analysis, and array-CGH along with conventional histologic analysis. This model retained the histopathological features and molecular profile of the original tumors and showed the clinico-pathological heterogeneity of bladder cancer as well. Also, the concept of co-targeting PI3K and MAPK signaling for certain types of bladder cancer were initiated in pre-clinical studies using PDX models [117]. PDX models derived from patient tumor tissues tend to reflect the biological characteristics of human tumors more precisely than tumor cell lines. Fichtner *et al*. [118] reported that tumor cell lines may lack similarity to the original tumor and may not mimic the exact clinical situation. Additionally, tumor cells in culture are devoid of stromal and endothelial elements [119]. Thus, to cope with the microenvironment, these tumor cells undergo genetic and epigenetic changes that occur gradually over serial passages resulting in a phenotype different from the original tumor [120]. Moreover, PDX models are representative of the original tumor heterogeneous phenotype compared with cell lines and these models. However, some of the limitations of the use of primary human tissue xenografts include (i) patient-derived tumors may not establish a xenograft [121], (ii) Interference of mouse stroma with human stroma [121], (iii) tumor take rate may be as low as 35% [89, 122], (iv) time taken for tumor establishment may be long (∼4–5 months), and (v) heterogeneous tumor tissue transplants may require a high number of replicates for statistical validation. Nevertheless, once PDX models are fully validated, it may have several clinical applications, including screening of multiple therapeutic agents simultaneously and interpreting the mechanisms of resistance. This will contribute towards turning molecularly targeted therapy into personalized cancer therapy.

### Hollow fiber (HF) model

This model utilizes semipermeable biocompatible fibers that can be loaded with cancer cells and implanted surgically in animals, which can be treated by chemotherapeutic agents [123–126]. Moon *et al*. [127] filled polyvinylidene fluoride (PVDF) hollow fibers with human bladder cancer cell lines (CRL2742, 253JP, SW1710, HTB9) and surgically implanted these fibers subcutaneously and intraperitoneally into athymic nude mice. Subsequently, these mice were treated with gemcitabine, cisplatin, paclitaxel and after 6 days these fibers were recovered to determine cell viability. Although tedious, it is a cost-effective screening method because multiple cell lines can be evaluated for drug cytotoxicity within a short duration (assay time < 2 weeks).

### Monitoring and evaluation of animal models for bladder cancer

Methods for tumor growth evaluation and therapeutic effects in animal models depend on the model system used. In subcutaneous tumors, measurement of subcutaneous tumor mass and imaging of tumor cells labeled with a luminescent or a fluorescent tag are instrumental for monitoring tumor growth and evaluation of diagnostic and responses summarized in? Detection of orthotopic and spontaneous urethral tumors cannot rely only on palpable bladder mass, weight loss, and urinalysis. A transurethral mini cystoscope is used as a non-invasive method for detecting and monitoring superficial tumors [129]. However, similar to white light cystoscopy used in the clinic, it is unreliable for differentiation between low- *vs* high-grade cancer. It can neither assess level of invasion or CIS nor differentiate CIS from inflammation [130]. The combination of mini trans-urethral cystoscopy with optical imaging modalities and intravesical injection of fluorescent or photosensitive dyes as fluorescein or proto-porphyrin precursors significantly improved the diagnostic value of mini-cystoscopy [130]. Other conventional imaging methods in animal studies include abdominal ultrasound, positron emission tomography-computerized tomography (PET-CT) and magnetic resonance imaging (MRI) can be used for early tumor detection as early as 14 days after tumor inoculation in mice [131]. High frequency and high resolution intravesical ultrasound (HRUS) were recently reported to demonstrate high sensitivity in monitoring tumor growth in orthotopic bladder cancer mouse model as a rapid, efficient and comparatively inexpensive imaging modality [132, 133]. Chan *et al*. utilized targeted contrast enhanced micro US imaging that enabled detection of vascular changes during bladder cancer development and progression [89]. Photoacoustic imaging (PAI) is a hybrid imaging modality that couples optical and ultrasound imaging in real time [134]. In this technology, biological tissue is exposed to a short laser pulse (nano-second) that is absorbed by hemoglobin and other chromophores in tissue resulting in thermo-elastic expansion, which produces broadband pulses (MHz) of acoustic energy. These pulses propagate to the tissue surface and are detected by an array of ultrasound transducers [135]. As ultrasound scattering is two to three orders of magnitude weaker than optical scattering in biological tissues [136], PAI can provide a better resolution than optical imaging for depths greater than 1 mm. In a recent study, three non-invasive optical imaging modalities including, BLI, HRUS and PAI were combined to increase sensitivity and improve accurate quantification of tumor growth *in vivo*. This multi-modality evaluation technique provided significant anatomical, molecular, and functional information [137]. Intensity-focused HIFU uses high-intensity ultrasound waves to precisely treat only the solid malignant tumors with minimal damage to surrounding tissue. HIFU ablation leads to coagulative thermal necrosis and cavitation damage due to absorption of ultrasound energy during transmission in tissue [87]. Imaging and diagnostic approaches described above are valuable means of non-invasive tumor evaluation, specially monitoring response to therapeutics in the live animal. These methods are often coupled and validated with biomarker validation from biological samples in the live animals or with histology and immunohistochemistry from bladder tissues dissected after animal euthanasia [137–139].

Bioluminescence (BLI) is another commonly used optical imaging modality for both *in vitro* and *in vivo* non-invasive monitoring of molecular and cellular activities. BLI is considered to be a far superior imaging methodology compared with fluorescence, as BLI does not need excitation and avoids the auto-fluorescence background signal and can be combined with other modalities as theranostic approach [140]. Jäger and colleagues combined BLI with percutaneous ultrasound-guided injection of luciferase-tagged cells bladder cancer cells into the anterior bladder wall. They monitored xenograft growth and perfusion *in vivo* longitudinally during therapy and were able to inject therapeutic agents directly into the tumor under ultrasound guidance [103].

For preclinical molecular assessment, the availability of linked color markers in lentiviral systems proved to be valuable tools for phenotypic analyses and metastasis tracking. This facilitates monitoring the dynamic changes in fluorescent signals, and drug responsiveness can be noninvasively tracked in host animal [141]. Fluorescence Imaging (FLI) is one of the new imaging tools, commonly used to monitor *in vivo* processes with reporters such as green fluorescent protein (GFP), red fluorescent protein (RFP) and near-infrared proteins [141, 142]. More fluorescent probes in the infrared (IR) spectrum have been developed to either tag tumor cells, or generate reporter mice to combine non-invasive imaging, tumor size monitoring and the response to therapy, along with tracking the expression of genes or molecules in specific cell types [122, 143–147]. Recently, an optimized CRISPR-Cas9 based light-inducible gene expression system in bladder cancer cell was developed [148] to control *Tp53* gene expression in a dose-dependent manner and inhibit proliferation of cancer cell by modulating expression of p53 gene [148].

## CONCLUSIONS AND PERSPECTIVES

Mouse models of urothelial cancer of the bladder present useful tools for advancing the early diagnosis of the disease, implementing tests for high risk population for primary prevention and early diagnosis and stratification of patients for a given therapy. Mouse models that represent the natural history of the disease provide the advantage of studying the early premalignant stages of the disease and allow for capturing of key biological and molecular features of human cancers that are not clinically encountered. They can be exploited not only for identification of early diagnostic biomarkers, but also for identification of biomarkers related to specific genetic factors associated with the human disease. The use of transgenic and genetically-engineered mouse models can help recognize the challenges associated with modelling tumor heterogeneity, tumor-stromal interactions and the contribution of the stromal compartment and the immune system to disease progression. Furthermore, the use of transplantable models (syngeneic and xenografts) can be combined to carefully study the events in the multistep cascades of cancer progression, invasiveness and metastasis. Combining multiple models together with imaging technologies will enable the generation of robust models that can further our knowledge of the pathobiology of the disease and allow for the discovery and validation of novel diagnostic and prognostic biomarkers and personalized therapies.
